# Co-Operativity between MYC and BCL-2 Pro-Survival Proteins in Cancer

**DOI:** 10.3390/ijms22062841

**Published:** 2021-03-11

**Authors:** Walter Douglas Fairlie, Erinna F. Lee

**Affiliations:** 1 Olivia Newton-John Cancer Research Institute, Heidelberg, VIC 3084, Australia; doug.fairlie@onjcri.org.au; 2 School of Cancer Medicine, La Trobe University, Melbourne, VIC 3084, Australia; 3 Department of Biochemistry and Genetics, La Trobe Institute for Molecular Science, La Trobe University, Bundoora, VIC 3084, Australia

**Keywords:** apoptosis, MYC, BCL-2, BH3-only, BH3-mimetic, pro-survival

## Abstract

B-Cell Lymphoma 2 (BCL-2), c-MYC and related proteins are arguably amongst the most widely studied in all of biology. Every year there are thousands of papers reporting on different aspects of their biochemistry, cellular and physiological mechanisms and functions. This plethora of literature can be attributed to both proteins playing essential roles in the normal functioning of a cell, and by extension a whole organism, but also due to their central role in disease, most notably, cancer. Many cancers arise due to genetic lesions resulting in deregulation of both proteins, and indeed the development and survival of tumours is often dependent on co-operativity between these protein families. In this review we will discuss the individual roles of both proteins in cancer, describe cancers where co-operativity between them has been well-characterised and finally, some strategies to target these proteins therapeutically.

## 1. The BCL-2 Family of Proteins Regulate Apoptosis

**Figure 1:**
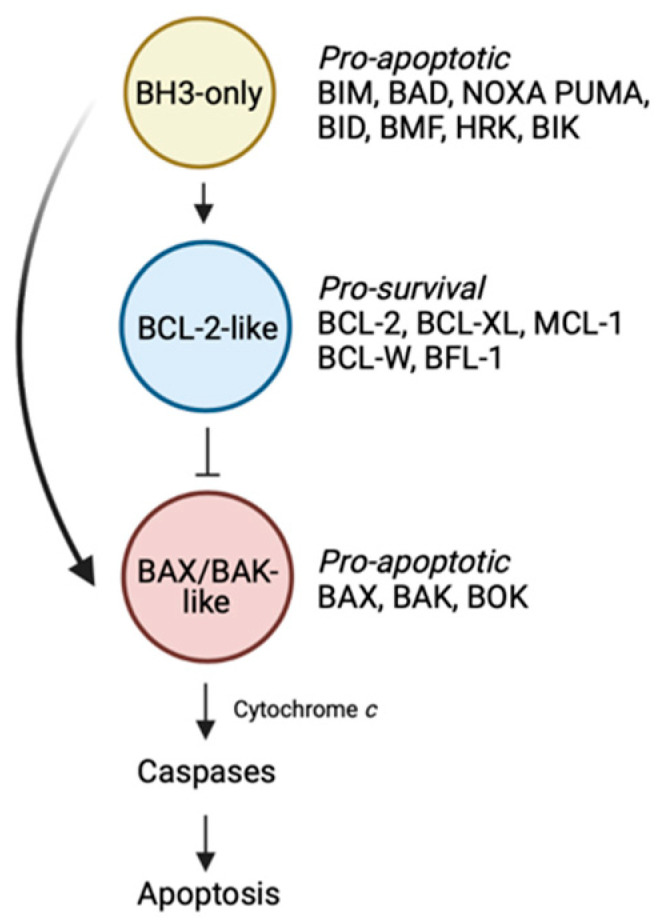
The BCL-2-regulated apoptotic pathway. The BH3-only proteins trigger the apoptotic cascade by either binding the BCL-2-like pro-survival proteins, displacing the BAX/BAK-like proteins, or alternatively in the case of certain members (e.g., BIM, BID, PUMA), by directly engaging and activating BAX/BAK. These events lead to BAX/BAK oligomerization followed by mitochondrial outer membrane permeabilisation, caspase activation and death. Figure created with Biorender.com.

There is a delicate balance within cells that dictates whether they live or die, and this exigency must be met to maintain a disease-free state. The BCL-2 family is an important group of proteins that holds tight rein on this counterpoise of whether a cell survives, or alternatively, is eliminated by the process of apoptosis (Figure 1) [1].

Within the BCL-2 family, there are proteins that promote cell death and others that enable cell survival. Of the cell death promoters, there are two sub-families. The first comprise the “BH3-only” proteins which trigger the apoptotic cascade [1]. Upon receipt of a death stimulus, these BH3-only proteins are transcriptionally upregulated, or post-translationally modified, enabling them to act on downstream pro- and anti-apoptotic family members, thereby initiating the slippery slide to cell death. In mammals, there are eight main members including BIM, PUMA, BID, NOXA, BID, BAD, BMF and HRK. The second sub-family of death-promoting molecules are the “BAX/BAK-like” proteins [1]. This family which includes BAX, BAK and BOK are the downstream effectors of the family. These multi-domain proteins, once activated, oligomerise to form pores resulting in mitochondrial outer membrane permeabilisation. A consequence of this event is the release of apoptogenic factors such as cytochrome *c* from the mitochondria into the cytosol, leading to activation of the cellular demolitionists, the caspases.

The last faction within the BCL-2 family are the “BCL-2-like” pro-survival proteins [1]. In mammals, there are five members: BCL-2 itself, BCL-XL, BCL-W, MCL-1 and BFL-1. In healthy cells, pro-survival proteins can be found in heterodimeric complexes with BAX or BAK preventing their oligomerisation [2]. Alternatively, pro-survival proteins can also bind to, and inhibit the ability of upstream BH3-only proteins to directly activate and induce oligomerisation of the BAX/BAK sub-family [2].

The rules of engagement describing the differential binding specificities of the pro-apoptotic proteins and pro-survival proteins are now well-defined and contribute to the highly tuned and ordered network of protein–protein interactions that dictate cell survival [3,4,5]. Serendipitously, the importance of the natural binding specificities that exist between the opposing factions of the BCL-2 family proved critical to the design of anti-cancer therapeutics targeting this pathway, which will be discussed later.

### 1.1. The Role of Pro-Survival BCL-2-Like Proteins in Tumourigenesis

Resisting cell death is a well-defined hallmark of cancer [6]. It is intuitive to think that aberrantly high levels of proteins that promote cell survival, or on the other hand, insufficient pro-death protein activity, can lead to tumourigenesis. In line with this, the identification of genetic lesions in human cancers [7,8,9], together with the use of genetically engineered mouse models [10,11] that lead to both these states, provided convincing evidence supporting an important role for members of the BCL-2 family in cancer.

The founding member of the BCL-2 family is BCL-2 itself. The gene was first identified during the heyday of oncogene discovery through the study of chromosomal rearrangements. Indeed, BCL-2 was discovered by mapping a t(14;18) translocation in an acute B lymphocytic leukaemia (ALL)-derived cell line [8]. The same chromosomal translocation was later observed in other haematological malignancies including 80% of follicular B-cell non-Hodgkin’s lymphomas (FL) [12,13,14], 20% of diffuse large B-cell lymphoma (DLBCL) [14], and more rarely in B-cell chronic lymphocytic leukaemia (CLL) (about 2–4% of cases) [14,15,16]. The gene for BCL-2 was cloned by three separate groups from FL, DLBCL and normal cells [8,12,17,18,19]. It was subsequently discovered through molecular analysis, that the translocations in these different diseases, though cytogenetically identical, arise via differing mechanisms [20]. However, despite these molecular differences, the shared outcome of this translocation event was the placement of the *BCL2* gene under the control of the immunoglobulin heavy (IgH) chain gene enhancer, resulting in the aberrant high-level constitutive expression of BCL-2.

Importantly, it soon came to light that it was this high level of BCL-2 expression, and not the presence of the t(14;18) chromosomal translocation, that was important in tumourigenesis [21,22]. High levels of BCL-2 expression, comparable to that observed in t(14;18)-containing haematological malignancies, is also seen in FL [23], CLL [24,25], DLBCL [26], multiple myeloma (MM) [27] and mantle cell lymphoma (MCL) [28] despite the absence of the t(14;18) translocation. Multiple mechanisms have now been reported by which deregulation of BCL-2 expression can occur. These include the deregulated expression of BCL-2 transcriptional activators [29], somatic mutations in the BCL-2 promoter region [29], loss of microRNAs that negatively regulate BCL-2 [30,31,32,33], *BCL-2* gene amplification or its transcriptional upregulation through constitutive activation of the NF-κB pathway [34]. Notably, this phenomenon is not restricted to just blood cancers but also extends to solid cancers such as lung [35], prostate [36], liver [37], and breast carcinomas [38] in which high levels of BCL-2 expression is observed even in the absence of *BCL-2* gene rearrangements.

Accordingly, detection of the t(14;18) translocation has little prognostic significance. Instead, it is the high levels of BCL-2 protein expression that serves to predict poor prognosis, reduced overall and disease-free survival, and recurrence in cancers [39]. For example, enhanced expression of BCL-2 is associated with the development of androgen-refractory prostate cancer [40], whilst in CLL, higher expression of BCL-2 is an adverse prognostic feature [41]. High BCL-2 expression also dictates poorer patient outcome following standard chemotherapy [22,39,42,43,44]. However, it should be noted that the role of BCL-2 expression as a prognostic marker also does not always hold up [35,45,46] such as in studies of advanced head and neck carcinoma and bladder cancer [47,48]. In fact, in some cases, BCL-2 expression correlates with improved clinical outcome, for example in patients with Estrogen Receptor (ER)- and Progesterone Receptor (PR)-positive breast cancer who received adjuvant endocrine therapy [49,50].

### 1.2. BCL-2—Defining a New Class of Oncogenes

It became increasingly evident that overexpression of BCL-2 contributes to tumourigenesis. The question then was—how does this occur? The discovery of BCL-2 not only identified a novel oncogene but perhaps more importantly, defined a paradigm-shift in what we understood about oncogenes. The landmark study using cytokine (IL-3)-dependent cell lines in culture demonstrated that, in contrast to all other known oncogenes at that time which promoted deregulated proliferation, overexpression of BCL-2 instead protected cells against death by apoptosis following cytokine deprivation [9]. These findings underlined that it is not just defects in the control of cellular proliferation that can promote tumourigenesis, but that defects in cell death leading to unwanted survival, were also important. Furthermore, the studies on the anti-apoptotic properties of BCL-2 defined for the first time that distinct genetic programs control cell proliferation versus cell survival. Now, apoptosis is widely accepted as a prominent tumour-suppressive function and that the inhibition of this pathway (such as through the overexpression of pro-survival proteins) is a key hallmark of cancer and drug resistance [6].

Subsequent to the discovery of the function of the first BCL-2 family member, additional members of the family were identified largely based on sequence conservation of up to four regions of homology known as Bcl-2 homology (BH) domains. These included four additional pro-survival members namely BCL-XL, BCL-W, MCL-1 and BFL-1, all of which have since been shown to contribute to the survival of various cancers.

### 1.3. Other Pro-Survival Members of the BCL-2 Family and Their Contributions to Tumourigenesis

A comprehensive study examining somatic copy-number alterations (SCNAs) in frequently altered genomic regions in over 3000 cancer specimens, from broadly 26 histological types, identified key genes with potential causal roles in tumourigenesis [51]. Amongst the gene families enriched included members of the BCL-2 family. Consistent with the notion that evasion of cell death is a hallmark of cancer [6], the pro-survival proteins BCL-XL and MCL-1 were found in amplification peaks when compared to non-cancerous samples, whilst pro-apoptotic proteins such as BOK and PUMA were identified in deletion peaks.

#### 1.3.1. MCL-1

The gene for MCL-1 was discovered as an early response gene induced during the differentiation of a human myeloid leukaemia cell line [52]. Strikingly, one of the most common focal amplifications (1q21.2) detected (in 10.9% of all cancers) contains the *MCL-1* gene. Amplification of this region has been previously reported in lung adenocarcinoma [53,54], breast cancer [51], and melanoma [55]. Validation that amplified MCL-1 is the contributing factor in cancers came when knockdown of *MCL-1* led to a significant reduction in cell growth in *MCL-1*-amplified cell lines, but not *MCL-1*-unamplified lines [51]. Like BCL-2, multiple mechanisms exist to give rise to high levels of MCL-1. These include microRNA deregulation, where mir-29b downregulation leads to increased MCL-1 expression in CLL and cholangiocarcinoma [31,56] or upregulation of MCL-1 via deregulated external stimuli such as through vascular endothelial growth factor (VEGF) or Interleukin-6 signalling in multiple myeloma and cholangiocarcinoma [57,58]. High levels of MCL-1 are also found in blood cancers such as CLL, ALL, FL [59,60,61] and is associated with chemoresistance and disease severity [59,60,61,62].

#### 1.3.2. BCL-XL

Like MCL-1, BCL-XL is one of five genes encompassed in a region of amplification (on 20q11.21) [51] that has been reported in lung cancer [63], giant-cell tumour of bone [64] and embryonic stem cell lines [65,66]. Using the same approach by which MCL-1 was validated as the key survival factor for *MCL-1*-amplified cancer cells, BCL-XL was knock-down in cell lines in which BCL-XL was amplified, resulted in a pronounced reduction in viability [51]. As with most pro-survival members of the BCL-2 family, elevated levels of BCL-XL have also been found in a number of different blood cancers. High levels of BCL-XL have been reported in multiple myeloma and correlates with increased chemoresistance, although BCL-2 appears to feature more prominently in this cancer type [27,67]. However, its role as a predictor of clinical outcome for multiple myeloma remains debatable [68]. Expression of BCL-XL has also been implicated in the development and chemoresistance of Bcr/Abl+ chronic myelogenous leukaemia as this pro-survival protein is a transcriptional target of signal transducer and activator of transcription (STAT) 5 which is involved in the anti-apoptotic activity induced by Bcr-Abl-mediated leukemogenesis [69]. In a study using cancer genomics data sets derived from over 5000 tumour samples from 20 cancer studies to identify cancer types with significant amplification of BCL-XL (The cBio Cancer Genomics Portal [70]), both colorectal cancer and cervical cancer demonstrated the highest percentage of BCL-XL gains and amplifications across all cancer types analysed [71]. In particular, BCL-XL has been identified as a driver in colorectal tumourigenesis and cancer progression [72].

#### 1.3.3. BFL-1

Unlike its other more well-characterised siblings, the role of BFL-1 (or A1 in mice) in tumourigenesis is perhaps not as far advanced. However, multiple studies are now pointing to an important role for it in tumour progression. Elevated levels of BFL-1 have been observed in B-cell CLL, AML, MCL and primary mediastinal large B-cell lymphoma [73,74,75,76] and contribute to chemoresistance and disease progression. For example, in B-cell CLL patients, BFL-1 levels were significantly higher in patients with no response to last chemotherapy as compared to patients that responded, or who had not required treatment [76]. Likewise, high BFL-1 expression correlated with more severe cases of CLL, indicating a potential prognostic role for BFL-1 [74]. Aberrant BFL-1 expression has been documented in various non-haematological malignancies including stomach [77] and breast cancers, especially in advanced breast cancer suggesting an association with later and more severe disease stages [78,79]. Overexpression of BFL-1 has also been implicated in melanoma cell survival although results have varied between studies as to its absolute essentiality for survival [80,81,82,83,84,85].

#### 1.3.4. BCL-W

Perhaps the pro-survival member that has received the least attention in the context of tumorigenesis is BCL-W. Despite relatively few studies, BCL-W has been found to be significantly overexpressed in a wide range of human B-cell lymphomas, including Burkitt Lymphoma (BL), diffuse large B-cell lymphoma (DLBCL) and Hodgkin lymphoma patient samples and cell lines [86,87,88]. As with BCL-2 and MCL-1, for which deregulation of the microRNA control of their expression has been described in cancer, miR-133b deregulation has been observed in bladder cancer, colorectal carcinoma and lung cancer leading to BCL-W overexpression in these cancers [89,90,91].

### 1.4. Pro-Apoptotic Proteins in Tumourigenesis

Whilst not a key focus of this review, it should also be noted that loss-of-function or deregulation of pro-apoptotic members of the family have also been implicated in tumourigenesis. For example, *Bak* mutations have been reported in human gastric and colorectal cancers, predisposing those patients to the development of these gastrointestinal malignancies [92]. Loss-of-function mutations in BAX have been detected in haematological and colorectal malignancies [93,94] and downregulation of BH3-only proteins such as BIM due to various mechanisms such as homozygous deletion or promoter hypermethylation has been observed in MCL, DLBCL and BL [7,95,96].

The oncogenic potential of dysfunctional apoptosis is inarguable. Overexpression of pro-survival proteins likely promotes tumourigenesis by keeping cells that are otherwise programmed to die, alive. In so-doing, this enhanced resistance to dying increases their risk of acquiring additional oncogenic mutations, including ones that deregulate the control of cellular proliferation such as MYC.

## 2. MYC: A Master Transcription Factor

MYC refers to a family of three proto-oncogenes (*c-MYC,* MYC; *n-MYC,* MYCN, *l-MYC*, MYCL) that were first identified by their homology to *v-MYC*, an avian myelocytomatosis MC29 retrovirus gene capable of cellular transformation [97,98,99,100]. The MYC oncoproteins are all transcription factors consisting of an N-terminal transregulatory domain and a C-terminal DNA-binding and dimerisation domain. The N-terminal region is not particularly well-conserved between family members apart from six short regions of homology (“MYC boxes”) that enable association with different binding partners, or which can be modified in different ways, leading to diverse functional outcomes including regulation of MYC stability/degradation, chromatin remodelling, histone acetylation and enhancing promotor affinity. The C-terminal region is, in contrast, highly conserved between MYC proteins and comprises basic (b), helix-loop helix (HLH) and leucine-zipper (ZIP) (collectively “bHLH-Zip”) subdomains. The HLH and ZIP domain enable MYC to associate with the related bHLH-Zip protein, MAX, forming obligate heterodimers required for binding to enhancer-box (E-box) sequences within the promotors of MYC target genes [101,102,103], particularly those located proximal to CpG islands [104,105]. However, where MYC-MAX dimers bind is concentration dependent, and also includes enhancers, degenerate E-boxes, as well as some sequences without an E-box [106].

Estimates of the number of genes regulated by MYC vary, though is certainly in the thousands [107,108,109,110], and could perhaps include every active gene within a cell [111,112]. This is in part due to the abundance of E-box motifs within the genome, as well as the aforementioned capacity to bind outside of these sites. Not surprisingly, MYC has been implicated in regulating essentially every basic cellular function including cell cycle progression, differentiation, growth, metabolism, DNA replication and apoptosis as well as more specific functions which, amongst many others, includes cell adhesion, epithelial-mesenchymal transition and angiogenesis, all of which are all important in cancer metastasis.

As the range of these functions and the literature describing them is so vast, we will confine any detailed discussion on how MYC regulates these processes to just the one that is most relevant to this review, namely apoptosis, which for obvious reasons is the predominant role for MYC and its co-operativity with BCL-2 (see Section 3 and Section 4 below). Nevertheless, it is informative to first provide some general background on MYC regulation/deregulation and how it contributes to tumourigenesis.

### 2.1. Mechanisms of MYC Activation and Its Deregulation in Cancer

*MYC* is referred to as an “*immediate early*” gene. The MYC protein, is generally present at very low levels in normal or quiescent cells but is rapidly induced following mitogenic signals transduced via multiple cellular pathways including MAPK, WNT, NOTCH, and PI3K that are also frequently deregulated in cancer [113,114,115,116,117,118,119]. MYC-target gene mRNAs, as well as long non-coding RNAs, tRNAs and microRNAs, are transcribed by all three RNA polymerases (Pol I, Pol II, Pol III) [120,121,122].

In cancer, MYC levels are greatly enhanced, and in some cases, by orders of magnitude [112]. This is a consequence of a number of distinct possible mechanisms. The first of these to be discovered was the upregulation of v-MYC by insertion of a retroviral promoter by another avian (leukosis) retrovirus [123,124]. Importantly, MYC is unlike many, if not most, other oncogenes in that it does not need to be mutated to unleash its oncogenic potential. Rather, increased expression alone is sufficient to promote tumorigenesis. Most common of the mechanisms by which this occurs is gene amplification where increased MYC copy number can result in increased expression [125]. In some human cancers, the *MYC* loci can also be disrupted by chromosomal translocations. This occurs in essentially all Burkitt’s lymphoma where the MYC gene on chromosome 8 is translocated into one of several heavy and light chain immunoglobulin loci on chromosome 14 (i.e., t(8:14) translocation), driving high levels of MYC expression [126,127]. Other important mechanisms that lead to high-level MYC expression include transcriptional upregulation due to deregulated upstream signalling pathways such as WNT, PI3K and NOTCH [115,116,119], stabilisation of *MYC* mRNA [128], increased export of MYC from the nucleus leading to increased MYC translation [129,130], reduced MYC degradation via loss of the ubiquitin ligases SCF^Fbw7^ and SCF^Skp2^ or mutations within different regulatory regions, especially at threonine 58 in the MYC degron (an interaction site for SCF^Fbw7^) [128,131,132,133,134], or stabilisation of the protein by phosphorylation (e.g., by ERK or GSK3) [118,135].

### 2.2. Mechanisms of MYC Deregulation in Promoting Tumourigenesis

Apart from early reports on the cell-transforming capacity of *v-MYC*, there are multiple lines of evidence supporting the importance of deregulated MYC expression in driving tumour development and progression. These have been reviewed extensively over the years but include indirect observations, as well as direct connections with tumourigenesis in experimental animal models [136]. For example, as described above, the levels of MYC are often elevated in tumours relative to non-cancerous tissue of the same origin. In patients, high MYC levels are often also associated with poor prognosis [137,138,139]. Cells that overexpress MYC take on characteristics of tumour cells, proliferating and growing more rapidly, whilst ablation of MYC results in the opposite effect [140]. Similarly, transgenic mouse models have shown that MYC overexpression results in increased tumourigenesis, whilst deletion or reduction in MYC levels, or its inhibition following expression of an engineered dominant negative mutant, can eliminate tumour development in certain models [141,142,143].

Although the combined evidence for the role of MYC in cancer is compelling, the mechanisms by which high levels of MYC drive tumourigenesis are more contentious [106]. Whilst it is clear increased MYC levels can cause cells to cycle more rapidly and to induce quiescent cells to renter the cell cycle, even in the absence of growth factors [144,145,146], MYC itself is actually a relatively weak transcription factor with expression of many specific target genes often increasing by less than two-fold [147,148], even when MYC levels are significantly upregulated. Furthermore, which specific genes are important for cellular transformation has yet to be conclusively established though changes in the expression (upregulation and suppression) of at least 40 MYC target genes have been implicated [149]. It has also been proposed that the increase in global (rather than specific) RNA levels due to increased genome-wide transcription could lead to oncogenesis. Increased MYC might also drive the formation of MYC-MAX dimers and increased affinity for, and occupation of specific gene promoters [150]. More recently, it has also been argued that target gene-independent functions of MYC associated with its vast interactome, including promotion of transcription termination upon stalling RNA Polymerase II, and its ability to coordinate transcriptional elongation with DNA replication and cell cycle progression, are likely critical factors in the mechanisms by which MYC promotes tumorigenesis [106].

Regardless of the specific details of these mechanisms, one process that has been inextricably linked to MYC overexpression is the induction of apoptosis. Whilst it might appear counterintuitive that increased MYC can lead to increased apoptosis in the context of tumourigenesis, the associated upregulation of oncogenic pro-survival proteins to counter this effect is a prominent feature of the development of some tumours. In the following section, we will discuss the roles of MYC in inducing apoptosis in more detail, and then review the mechanisms underlying the co-operativity between these two important signalling pathways in cancer.

## 3. MYC—A Driver of Apoptosis

In the early days, the oncogenic potential of MYC was classically attributed its ability to drive cell-cycle progression and the hyperproliferation of cells. Paradoxically, MYC expression in late passage fibroblasts is associated with tumours that grow less aggressively and with decreased ability to metastasise, as compared to for example RAS-expressing tumours, with cell loss by apoptosis commonly observed [151]. This ability of MYC to induce or sensitise cells to apoptosis, regardless of the phase of the cell cycle, was subsequently demonstrated in various cell types including factor-dependent myeloid cells, fibroblasts and self-reactive T-cells [152,153,154]. Furthermore, the level of MYC expression was shown to positively correlate with the extent of apoptosis induced both in vitro and in vivo [153,155]. Here, careful rheostat-like control of MYC levels demonstrated that a modest increase of MYC expression enhanced transformation, whilst robust expression led to significant apoptosis instead [155]. Notably, the domains on MYC that are responsible for conferring its apoptotic capacity overlap with regions of the protein required for its other well-accepted roles in transformation, sequence-specific DNA binding, MAX dimerisation and transcriptional activation [153]. Hence, whether the proliferation or apoptotic program is engaged is dependent on the operational threshold at which MYC triggers these distinct outputs, leading to dramatically distinct outcomes. This threshold is seemingly exquisitely dependent on the internal state of the cell and its microenvironment [155].

### 3.1. Mechanisms by Which MYC Induces Apoptosis

**Figure 2:**
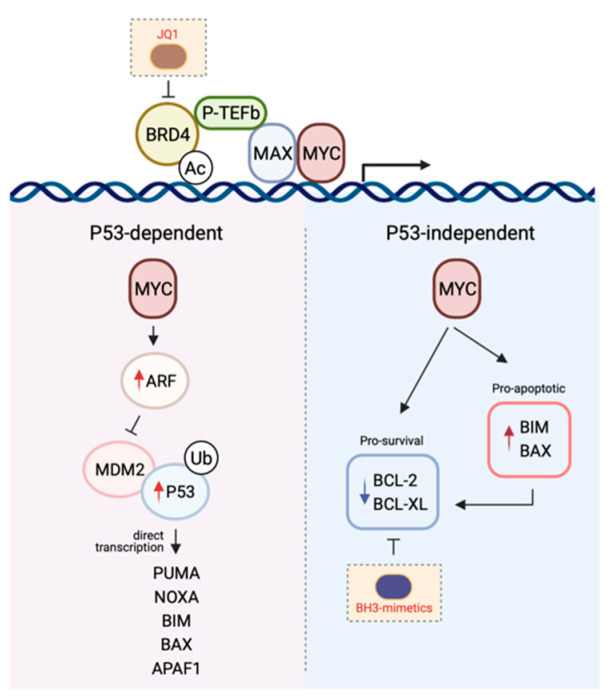
The mechanisms by which MYC induces apoptosis. The P53-dependent pathway to MYC-induced death is primarily mediated by the ARF-MDM-P53 axis following the upregulation of ARF expression by MYC. This leads to the stabilisation of P53 and the induction of P53 pro-apoptotic target genes. In contrast, the P53-independent pathway to MYC-induced apoptosis is reliant on the direct transcription of pro-apoptotic genes or the repression of pro-survival protein expression. Given the cooperativity between MYC signalling and the BCL-2-regulated apoptotic pathway in promoting tumourigenesis, combining drugs targeting both arms (e.g., MYC with JQ1, BCL-2 pro-survival proteins with BH3-mimetics) is a promising therapeutic avenue (see Section 5). Figure created with Biorender.com.

This latent or intrinsic tumour suppressor function of MYC, mediated by its ability to induce apoptosis has been heavily investigated. Moreover, the molecular mechanisms describing the crosstalk between MYC signalling and apoptosis induction is generally well-understood and can be broadly dichotomised into P53-dependent versus P53-independent mechanisms (Figure 2).

#### 3.1.1. P53-Dependent MYC-Induced Apoptosis

In a healthy cell, P53 is normally short-lived and found at low levels. However, following receipt of a stress stimulus or DNA damage, it is stabilised and accumulates in order to exert its inhibition on cell cycle progression or cell survival. The tumour suppressive role of the transcription factor P53 is attributed to its ability to induce apoptosis [156]. This occurs mostly by direct transcriptional activation of the BH3-only protein PUMA, and to a lesser extent NOXA [157,158,159,160,161]. In addition, P53 is also thought to regulate BIM expression, although it is less clear whether this occurs via indirect [162,163] or direct mechanisms of transcriptional regulation [164,165,166]. In addition to BH3-only proteins, both BAX and APAF1 have also been shown to be transcriptional targets of P53, though their participation in P53-mediated apoptosis is likely cell-type dependent [167,168,169].

Concurrent with P53 accumulation, expression of the P53-target gene MDM2 is also induced [170,171] which serves as a negative-feedback loop as MDM2 binds to, and targets P53 for proteasomal degradation, inhibiting its transcriptional activity [172,173,174]. Upstream of this P53/MDM2 node is the tumour suppressor ARF, which is perhaps the second most commonly deleted or mutated locus in cancer, behind P53. The ARF protein binds to MDM2, inhibiting MDM2-induced P53 degradation and transactivational silencing, and can do so as a ternary complex [175,176,177,178]. Alternatively, ARF has also been proposed to inhibit MDM2-mediated nuclear export of P53 into the cytoplasm hence leading to the stabilisation of the latter [176,179].

The importance of the ARF-MDM2-P53 axis in MYC-induced apoptosis [180] is supported by several lines of evidence. Overexpression of MYC in cells leads to the induction of ARF expression and P53-dependent apoptosis, and the loss of ARF or P53 renders cells highly resistant to the deleterious effects of elevated MYC levels [181]. Consistent with this, in the face of MYC overexpression, wild-type cells that sustain P53 mutations and ARF hemizygous cells bearing loss of ARF are conferred a selective advantage enabling their continuous proliferation [181,182]. Perhaps the most elegant and convincing evidence was provided by studies carried out in the Eµ-MYC transgenic mouse model. Tumour latency in this mouse model is typically six-months prior to the onset of disease, with high levels of apoptosis detected in the B-lymphocyte compartment consistent with the induction of apoptosis due to high MYC levels [183]. However, in approximately half of the spontaneous tumours that do arise this model, inactivation of the ARF-MDM2-P53 axis, through either ARF (biallelic deletion), P53 loss of function (by mutation or biallelic deletion), or overexpression of MDM2, is observed [182]. In addition, ARF or P53 deletion markedly accelerated lymphomagenesis in the context of the Eµ-MYC transgenic mouse model [184]. These and other studies (which due to space limitations have not been included in this review) underscore the role of the ARF-MDM2-P53 axis in delivering the fatal blow induced by MYC overexpression.

#### 3.1.2. P53-Independent MYC-Induced Apoptosis

Intriguingly, high levels of MYC can still kill cells in the absence of P53, strongly suggesting that P53-dependent signalling is in fact not obligatory for MYC-induced apoptosis [185]. Logically, it makes sense that the MYC-induced apoptotic program has in place P53-independent mechanisms so that even in the context of P53 loss-of-function, which is a frequent event in oncogenesis, cells still retain the capacity to protect themselves in the face of deregulated cellular proliferation. Not unexpectedly, though tellingly, the mechanisms enabling the P53-independent crosstalk between MYC and apoptosis is largely mediated by members of the BCL-2 family as a consequence of the transcriptional activity of MYC.

A key mediator implicated in MYC-induced apoptosis is the BH3-only protein BIM. Independent of P53 status, BIM expression is transcriptionally induced following binding of MYC to the BIM promoter, inducing BIM overexpression [10,186,187]. Perhaps the most convincing evidence supporting a role for BIM induction in mediating the tumour suppressive function of MYC was provided in experiments using MYC mutants incapable of inducing the expression of this pro-apoptotic protein. Here, mice transplanted with haematological cells expressing these MYC mutants succumbed to lymphomas more rapidly than their wild-type counterparts [186]. Notably, these mutants had no impact on the proliferative potential of MYC, further emphasising the importance of the pro-apoptotic capacity of this oncogene in tumour surveillance. Importantly, this observation has been seen in human Burkitt’s lymphoma where BIM expression is virtually absent in tumours carrying mutant MYC [186,188]. The role for BIM as a mediator of MYC-induced apoptosis extends beyond that seen in lymphomagenesis and has also been observed in multiple solid tissues [187].

In addition to BIM, MYC can also engage E-boxes in the *BAX* promoter to upregulate BAX expression and induce apoptosis [189]. Notably, apoptosis can still ensue following MYC overexpression in *BAX*^−/−^ cells, though not to the same extent as in the *BAX*^+/+^ control cells, suggesting other mechanisms are also involved in cell killing (e.g., via BIM-induced BAK activation). MYC has also been shown to repress both mRNA and protein expression of pro-survival members such as BCL-2 or BCL-XL [190,191], hence inhibiting the induction of apoptosis directly.

## 4. Cooperativity between Myc and the Bcl-2-Regulated Apoptotic Program in Tumourigenesis

### 4.1. Cooperativity between MYC and Elevated Pro-Survival Proteins

Deregulated MYC expression is a common event in tumour cells indicating that this is an essential step in tumourigenesis. It therefore makes sense for cells to have developed a built-in failsafe program to limit the resulting unchecked cell proliferation. The proliferative advantage conferred by MYC overexpression cannot be disentangled from its ability to induce apoptosis, and hence its deregulation should be lethal to a cell. It is thus reasonable to conclude that whilst MYC deregulation is an essential step in tumourigenesis, cells also need to acquire a secondary block in apoptosis signalling preventing their demise. The observation that heightened sensitivity to apoptosis caused by ectopic MYC expression is observed in premalignant cells, but not after malignant transformation provides evidence that tumour cells do acquire specific mechanisms to blunt the pro-apoptotic effects of MYC deregulation [192].

In the same way deregulated MYC alone does not lead to full malignant transformation, the overexpression of BCL-2 pro-survival proteins alone is also similarly insufficient. The t(14;18) chromosomal translocation leading to the deregulated expression of BCL-2 is now a well-established oncogenic hit, particularly in B-cell lymphomas. Landmark studies using transgenic mice to recapitulate this translocation, where the BCL-2 gene was linked to the immunoglobulin heavy-chain gene enhancer, resulted in the polyclonal expansion of B lymphoid cells, in particular immature and mature B cells, as well as Ig-secreting plasma cells, and enabled their prolonged survival in vitro [193,194,195]. However, tumour incidence observed in these mice was unexpectedly low, with only 5–20% progressing through, and only at an advanced age (one to two years), to a monoclonal lymphoma or plasmacytoma, instead of recapitulating the follicular lymphoma characteristic of the t(14;18) chromosomal translocation in humans. In addition, IL-3 dependent myeloid progenitor cells overexpressing BCL-2 failed to produce tumours when injected into mice [9,196]. Intriguingly, this translocation has been detected in healthy individuals with only a small number expected to develop lymphomas [197]. The slow progression to tumour manifestation in the Eµ-BCL-2 mice and the existence of the t(14;18) translocation in healthy individuals, strongly suggest that the overexpression of BCL-2 as a consequence of this genetic aberration can occur beyond the context of malignancy and/or hyperplasia and likely precedes other key oncogenic steps (such as secondary genetic aberrations) required for full neoplastic transformation.

Consistent with these notions, the progression of follicular lymphoma to a more aggressive intermediate or high-grade lymphoma occurs in the majority of patients where the transformed lymphomas retain the t(14;18) translocation but also acquire new chromosomal abnormalities [198,199]. Notably, a new translocation of the MYC gene into the immunoglobulin locus is observed in ~10% of such transformed lymphomas (so-called “double-hit” lymphomas) [200]. This observation extends to other cancer types too, for example non-Hodgkin lymphoma [201], germinal centre B-cell lymphoma [202], and acute lymphoblastic leukaemia [203,204] where concurrent activation of BCL-2 and MYC occurred leading to their elevated levels.

These clinical observations that BCL-2 and MYC cooperate in neoplastic transformation had in fact already been proven experimentally. Firstly, early in vitro studies demonstrated that BCL-2 and MYC cooperated to favour the growth of pre-B and B cells [9,205]. Further compelling results were then derived from mouse experimental models. Landmark studies in mice doubly transgenic for BCL-2 and MYC developed tumours much more rapidly than mice expressing either transgene alone [11]. In addition, almost 50% of the high-grade diffuse large-cell immunoblastic lymphomas that arose in the BCL-2 transgenic mice harboured rearrangements in the MYC gene [206,207]. Validation that a blockade of apoptosis due to the sustained overexpression of BCL-2 was a required step during MYC-driven tumourigenesis was shown in a mouse model of lymphoblastic leukaemia where removal of this BCL-2 reliance using an inducible system led to leukaemia remission and prolonged survival of the mice [208].

Mechanistically, this cooperativity between MYC and BCL-2 was proven to be due to the ability of BCL-2 overexpression in mitigating the apoptotic effects of deregulated MYC expression, without affecting MYC’s mitogenic function [209,210,211]. This interaction between MYC and BCL-2 described a novel mechanism for oncogene cooperation that differed from the well-accepted cooperativity between oncogenes such as MYC and activated RAS.

Since these landmark studies with MYC and BCL-2, cooperativity with MYC in the promotion of malignant transformation has since been shown to extend beyond BCL-2 itself, and applies to other pro-survival members of the BCL-2 family. For example, overexpression of MCL-1 in multiple hematopoietic lineages accelerated MYC-driven tumourigenesis [212,213] whilst high levels of BCL-XL cooperates with deregulated MYC to lead to plasma cell malignancies and highly malignant leukaemia [214,215,216]. Consistent with these observations in mice, the most frequent other focal SCNA in human cancers harbouring an amplification in either MCL-1 or BCL-XL was amplification of the region carrying MYC (in ~2/3 of these cases) [51].

### 4.2. Cooperativity between MYC and Endogenous Pro-Survival Proteins

The studies described above convincingly demonstrated that deregulated cell proliferation and impaired cell death are potently synergistic in tumourigenesis. However, these scenarios all involved the enforced overexpression of the pro-survival protein compartment, in particular as a consequence of a chromosomal translocation. The question therefore remained as to whether endogenous levels of the pro-survival proteins would be sufficient to sustain the malignant growth and survival of MYC-induced cancers. Intriguingly, deletion of endogenous BCL-2 itself did not reduce the incidence or delay the onset of Eµ-MYC lymphomagenesis [217], despite earlier studies demonstrating the critical role of overexpressed BCL-2 in mediating this oncogenic cooperativity. These findings suggested that during the genesis of MYC-driven lymphoma, the acquisition of pro-oncogenic hits takes place at a stage when BCL-2 is dispensable. As the mice bearing an Eµ-*Myc*/*Bcl-2*^−/−^ haematopoietic compartment only showed significant compromise in the survival of the mature B cell subset [217], this suggested that it is likely the pro-B and/or pre-B cells (or earlier progenitors) that are responsible.

The pro-survival proteins BCL-XL and MCL-1 were obvious candidates as factors enabling this sustained tumour growth as they are expressed at several stages of B lymphopoiesis and are critical to the survival of B lymphoid progenitors and/or precursors. It was subsequently shown that BCL-XL is essential for Eµ-MYC-induced lymphoma growth, but loss of this protein did not significantly impact the sustained growth of such tumours [218]. Instead, it is MCL-1 which appears to be the key factor driving the sustained growth of Eµ-MYC lymphoma and even the loss of a single allele was enough to lead to complete regression in 20% of tumours [219].

There is overwhelming evidence to demonstrate that endogenous or overexpressed BCL-2 pro-survival proteins contribute to oncogenesis by permitting the survival of nascent neoplastic cells for long enough such that other advantageous oncogenic mutations, for example in MYC, can be acquired. Whilst not a focus of this review, loss of effective cellular pro-apoptotic function (e.g., loss of BIM [10], or BAX [220]) can also lead to cooperativity with MYC to accelerate tumourigenesis. These studies provide strong evidence that inhibition of MYC-induced apoptosis is a key enabling feature of the cooperation between pro-survival BCL-2 proteins and MYC.

## 5. Therapeutic Strategies Targeting Bcl-2 and MYC

Given the importance of MYC and BCL-2 proteins in cancer, it is unsurprising that there has been considerable interest in the discovery of drugs that can target both these proteins. In this section we discuss efforts around the discovery of drugs targeting these proteins individually, and then how some of these have been applied in combination.

### 5.1. Drugs Targeting BCL-2 Proteins

Although there was considerable early interest in the development of antisense oligonucleotide strategies to reduce BCL-2 levels in tumour cells, and some such as Oblimersen sodium showed some promising activity in clinical trials [221,222], this approach has now been overtaken with the advent of small molecule direct inhibitors of BCL-2 proteins. When the first three-dimensional structures of BCL-2 proteins became available, especially those in complex with their natural ligands, the BH3 domains of pro-apoptotic proteins, they immediately suggested a potential strategy to develop small molecule drugs that could induce apoptosis through mimicry of this interaction [223,224]. Subsequent similar structures of all BCL-2 family pro-survival proteins in complex with BH3 domains revealed a common mode of binding whereby the helical pro-apoptotic BH3 domain engaged a long hydrophobic groove containing several small pockets that accommodated hydrophobic moieties projected from the ligand [225,226,227,228,229].

In 2005, the first small-molecule compound, ABT-737, capable of mimicking this interaction was reported [230]. This so-called “BH3-mimetic” drug bound to BCL-2, BCL-XL and BCL-W with low nanomolar affinity and, unlike other putative BCL-2 protein inhibitors described at that time, was able to potently induce mechanism-based (i.e., BAX/BAK-dependent) apoptosis in cell lines, and tumour regression in mouse xenograft models [230,231]. Subsequently, an orally bioavailable analogue, ABT-263 (“Navitoclax”) was developed with a similar pro-survival protein binding profile [232,233]. Due to its promising preclinical in vivo activity, Navitoclax entered clinical trials though the response rate was relatively low and dose-limiting thrombocytopaenia was observed [234,235]. This toxicity was an on-target side-effect of Navitoclax having high affinity for BCL-XL, a critical protein for platelet cell survival [236].

In response to this toxicity, an analogue of Navitoclax, namely ABT-199/Venetoclax that was more specific for BCL-2 was developed [237]. Venetoclax showed significant preclinical activity in BCL-2 driven haematological malignancies in vivo and due to its highly promising results in early phase clinical trials in patients with relapsed or refractory chronic lymphocytic leukaemia, was fast-tracked for approval in the USA, and subsequently approved in other countries for use in a variety of blood cancers, either as a single agent, or combined with other targeted therapies such as Rituximab, where results have been particularly impressive [238]. Trials of Venetoclax with other agents such as chemotherapy (e.g., Azacitidine, Decitabine) are also underway [239,240]. In addition, AstraZenca have developed AZD4320, a dual BCL-2/BCL-XL inhibitor. This is administered intravenously just once per week, and although some thrombocytopaenia is observed, this is transient and platelet levels return to normal within a week of administration [241]. More recently, a dendrimer-conjugated version of AZD4320, AZD0466, was developed resulting in an improved therapeutic index enabling the progression of this optimised candidate into clinical development [242]. Hence, this compound has significant potential for use in BCL-XL-driven cancers.

Following the development of Venetoclax, a number of other pro-survival protein-specific inhibitors were developed. These include several compounds specific for MCL-1 such as S63845, AZD5991 and AMG 174 [243,244,245] that show significant efficacy in vitro and in vivo, and some of these are currently undergoing clinical trials. Despite the dependence of platelets on BCL-XL, several potent BCL-XL-specific inhibitors have also been developed including WEHI-539 and A-1331852 [246,247].

**Figure 3:**
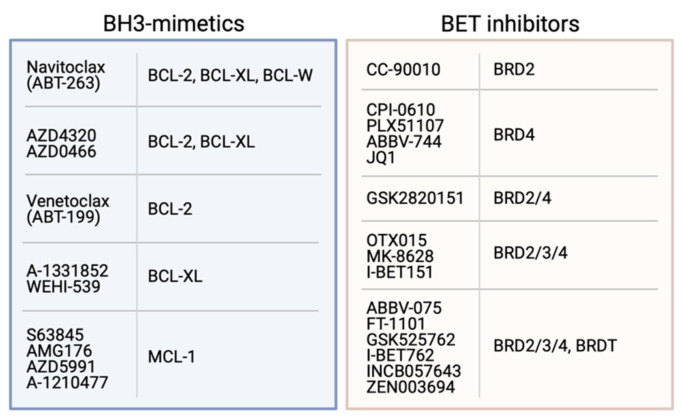
Examples of BH3-mimetics and their BCL-2 pro-survival proteins targets, and BET inhibitors with their BRD protein targets.

The availability of BH3-mimetics targeting most pro-survival proteins (Figure 3) has been enormously useful for studies determining the pro-survival protein dependency of many tumour cell types [247,248]. These studies have shown that although some haematological cancers are dependent only on BCL-2 or MCL-1 for their survival, many solid cancers are relatively resistant to most BH3-mimetics as single agents. However, potent killing is achieved when multiple pro-survival proteins are targeted, especially MCL-1 and BCL-XL [82,247,248,249,250,251]. Unfortunately, administration of compounds targeting both these proteins (e.g., using S63845 and A-1331852) resulted in acute hepatotoxicity and death in mice [250], although a recent study in melanoma showed MCL-1 and BCL-XL could be targeted with S63845 and ABT-263 if the dosing regimen was carefully controlled [251]. However, it is unclear whether such a strategy would ever be suitable for use in humans due to the potential risks involved. Notably, combinations targeting both MCL-1 and BCL-2 (e.g., S63845 and Venetoclax) have proven efficacious and safe in clinical trials in both haematological (e.g., AML, T-cell ALL, MCL) and solid cancers (e.g., melanoma) [252].

As there are potentially a very large number of solid cancers that could benefit from dual inhibition of MCL-1 and BCL-XL [248], a number of more tumour-specific approaches have been investigated. One way is to combine BH3-mimetics targeting one of these pro-survival proteins with targeted therapies, such as inhibitors of oncogenic kinases that work by modulating the expression of pro-apoptotic BH3-only proteins. For example, MCL-1 inhibitors (e.g., S63845 or AMG 176) have been combined with inhibitors against MEK, HER-2, B-RAF or EGFR and shown to induce a cytotoxic response in solid tumours such as breast, non-small cell lung cancer, lung adenocarcinoma and melanoma cells [243,253]. Perhaps the novel nanoparticle formulation of AZD0466, currently undergoing phase I clinical trials in haematological and solid cancers, will also provide a promising alternate means by which co-administration with an MCL-1 inhibitor can be achieved safely [242]. Another approach that is likely to have far greater specificity for tumour cells is an antibody-drug conjugate whereby A-1331852 is coupled to an antibody specific for cell surface proteins such as the epidermal growth factor (EGF) receptor that are frequently overexpressed in some cancers. Although such compounds have not yet been published, data available from patents suggest these molecules could be highly efficacious, though again it is unclear yet whether they can be safely combined with an MCL-1-specific inhibitor.

In MYC-driven cancers, combinations of BH3-mimetics with drugs that can act on MYC also have potential, and will be discussed below.

### 5.2. Drugs Targeting MYC

MYC is frequently referred to as being ‘undruggable’ [254]. This is largely a consequence of its mostly disordered structure which lacks well-defined pockets, grooves or other features that are usually targeted by small molecule drugs. Concern has also been raised about the potential unwanted side-effects associated with systemic inhibition of a transcription factor that regulates so many cellular processes. Although there are currently no ‘direct’ MYC inhibitors used in clinical practice, multiple approaches have been reported that can successfully modulate MYC activity in tumour cells, belying the undruggable label. Strategies that can regulate MYC activity typically fall into two major categories—those that can indirectly influence MYC levels in a cell and those that directly engage MYC or MYC/MAX dimers.

Despite the relatively featureless structure of MYC, a number of compounds have been identified capable of binding to it and inhibiting MYC/MAX dimerisation. Most of the earlier compounds such as IIA6B17, NY2267, 10058-F4 and 10074-G5 [255,256,257,258] have relatively low affinity for MYC or MYC/MAX dimers (i.e., >20 μM), though were capable of inhibiting cell growth in vitro, but with poor pharmacokinetic properties for in vivo application. More potent analogues of 10074-G5 have been developed (JY-3-094, 3jc48-3) though these have not been proven in vivo. More recently, the compounds Mycro3 and KJ-Pyr-9 were developed with significantly greater in vitro activity and anti-tumour activity in vivo, though none of these have yet to progress into the clinic. In parallel with the development of small molecule inhibitors, a peptidic approach has also been used to inhibit MYC activity. The best example is Omomyc, a mutated miniprotein based on the MYC bHLH-Zip domain [259,260]. This acts as a dominant negative protein of MYC and exerts its activity through multiple mechanisms including heterodimerisation with MAX as well as homodimerising which allows it to occupy E-boxes, but not transcribe the target genes due to the lack of the transactivation domain [259,260,261,262,263]. Omomyc has shown impressive anti-tumour activity in a number of transgenic mouse models [264,265,266,267] though it also has intrinsic cell-penetrating activity with in vivo activity in lung cancer models when administered via different routes (intranasally or intravenous) [268]. Hence, Omomyc also has significant potential for future clinical development.

A range of compounds that indirectly influence MYC expression in cells have been evaluated. Indeed, some of these have even advanced into early phase clinical trials. These include antisense oligonucleotides that degrade *MYC* mRNA (e.g., INX-3280) or phosphorodiamidate morpholino oligomers that inhibit MYC protein expression by preventing ribosomal assembly (e.g., AVI-4126/Resten-NG) [269,270]. Trials of both approaches were discontinued for various reasons despite some seemingly positive results. Similarly, siRNA have also been investigated using a range of nanoparticle formulations to improve siRNA stability and delivery [271,272,273,274,275]. Some of these have shown in vivo efficacy in different tumour models and a clinical trial of “DCR-MYC” showed some mechanistic activity, though was abandoned. Major efforts have also gone into development of compounds that can target and stabilise the G-quadraplex structure in the *MYC* promoter and its transcription. Some of these have been tested in clinical trials, though have also been discontinued [276].

Undoubtedly, the indirect approach to regulating MYC that has gained most attention in recent years is a class of drugs that can target the bromodomains of the bromodomain and extraterminal (BET) proteins BRD2, 3 and 4 (Figure 3). These compounds inhibit association of the BET proteins with acetylated histones on active chromatin, preventing recruitment of transcription factors and, thereby, blocking transcription of target genes, most notably, *MYC* [277,278]. Since the first BET inhibitor (BETi) JQ1 was reported, a number of other similar compounds have been developed, including some that are specific for particular bromodomains, bivalent BETi and BETi associated with proteolysis-targeting (i.e., PROTACs) moieties [279,280,281,282,283,284]. The BETi have shown efficacy in a wide range of haematological (e.g., AML, multiple myeloma, ALL) and solid cancers (e.g., NUT midline carcinoma, lung, breast, colon, prostate, brain etc) [285]. Despite evidence for the emergence of a number of resistance mechanisms to BETi [286,287,288], a number have undergone or are undergoing clinical trials, either as single agents or in combinations, for a large number of cancer types [285]. In general, these drugs have been shown to be tolerated albeit with some toxicities such as thrombocytopaenia and anaemia, though the outcomes from these have been mixed. None have yet to progress beyond trials and been approved for use.

### 5.3. Dual Targeting of MYC and BCL-2 Proteins

Given most cancers have deregulated MYC expression, and as a consequence, BCL-2 pro-survival proteins are often also deregulated to counter the pro-apoptotic effect of high MYC expression, there is a strong rationale for co-targeting of MYC and BCL-2 proteins in many cancers (Figure 2). This strategy to cancer treatment has gained significant momentum in the last five years with the advent of the BETi and BH3-mimetics (Figure 3) which are both suitable for in vivo studies and, moreover, are apparently safe and, in some cases, showing promising activity in patients.

Indeed, a wide range of BETi have now been tested together with various BH3-mimetics, though most studies have focussed on Venetoclax where enhanced responses from combining the drugs have been seen in vitro and in vivo in many haematological malignancies including T cell lymphoma, CLL, T cell acute lymphoblastic leukaemia, and diffuse large B cell lymphoma [289,290,291,292,293,294,295,296,297], and some solid tumours such as small cell lung cancer [298]. The dual BCL-XL/BCL-2 inhibitor Navitoclax was also shown to synergise with BETi in small cell lung cancer, colorectal cancer, glioma and B-cell lymphomas [299,300,301,302], whilst BH3-mimetics targeting MCL-1 enhance BETi activity in AML and melanoma [292,303].

Although BETi have the potential to act on multiple cellular pathways, their ability to co-operate with BH3-mimetics is generally associated with their capacity to down-regulate MYC expression. Nevertheless, several distinct mechanisms have been shown to underlie the synergy between BETi and BH3-mimetics. Although, BH3-mimetics act to neutralise any excess pro-survival proteins present within MYC-driven tumours, thereby lowering the threshold for apoptosis induction, the mechanism by which the drugs co-operate is somewhat more complicated. For example, BET inhibition has been shown to suppress miR17-92, a key post-transcriptional repressor of BIM expression [304]. Not surprisingly, BIM upregulation and the resultant increase in the formation of BCL-2/BIM complexes was observed in many studies following BETi treatment [290,292,296,298]. This primes cells to apoptosis, especially that induced by Venetoclax, which can displace any BIM bound to BCL-2 for activation of BAX and BAK [237]. Levels of BCL-2 are also generally decreased following BETi treatment [290,292,293,294,298,305], though notably, other pro-survival proteins including BCL-XL [292,298], MCL-1 [292] and BFL-1 [295] have been shown to be reduced in different cancer types, leading to a further reduction in the apoptotic threshold of the cell. In colorectal cancer cells, BETi treatment led to repression of MYC-driven expression of miR1271-5p, which in turn led to increased NOXA levels and inhibition of MCL-1, thereby enabling synergy with ABT-263.

## 6. Conclusions

Despite the importance of the connection between deregulated MYC and BCL-2 pro-survival protein expression, and that the mechanisms by which they co-operate in cancer have been known for over two decades, there are still no clinically approved co-treatments that target both proteins. This is largely due to the fact that it has taken many years to develop potent compounds capable of inhibiting BCL-2 pro-survival proteins and MYC. However, recent advances in the development of a number of BH3-mimetics with various specificities, and the success of Venetoclax in patients, means that one arm of this co-operativity can now be effectively disarmed. Although the development of similar directly acting MYC inhibitors has yet to have an impact in the clinic, the BETi that exert much of their activity by downregulation of MYC provide the most promising approach to date to tackle this critical second arm driving many tumours. Most encouraging are the numerous studies described above showing synergy between BETi and BH3-mimetics, including in clinically relevant patient-derived xenograft models [292,293], which showed such combinations are also safe. Nevertheless, there has yet to be a clinical trial examining such combinations. Hence, we expect it is only a matter of time before such combinations are explored in the clinic, with the hope they can provide benefit for the many cancers that depend on the co-operativity between MYC and BCL-2 proteins.

## Data Availability

Not appliable.
